# Risk of acute kidney injury in patients on concomitant vancomycin and piperacillin-tazobactam compared to vancomycin and other beta-lactam agents: a large multicenter retrospective cohort study from China

**DOI:** 10.1128/spectrum.00821-26

**Published:** 2026-07-06

**Authors:** Kunming Pan, Xingyun Hou, Jie Li, Ying Zhang, Yuzhu Wang, Chengchun Zuo, Ranyi Li, Xiaoqiang Ding, Qianzhou Lv, Xiaoyu Li

**Affiliations:** 1 Department of Pharmacy, Zhongshan Hospital Fudan University 92323, Shanghai, China; 2 Department of Pharmacy, Fudan University (Xiamen Branch), Zhongshan Hospital, Xiamen, Fujian, China; 3 Department of Pharmacy, Second Afiliated Hospital of Naval Medical University, Shanghai, China; 4 Department of Pharmacy, Tongji Hospital of Tongji University, Shanghai, China; 5 Department of Nephrology, Zhongshan Hospital Fudan University 92323, Shanghai, China; Zhejiang University School of Medicine Sir Run Run Shaw Hospital, Hangzhou, Zhejiang, China

**Keywords:** vancomycin, piperacillin-tazobactam, beta-lactam agents, acute kidney injury, major adverse kidney events

## Abstract

Acute kidney injury (AKI) associated with vancomycin (VAN) plus piperacillin-tazobactam (PTZ) remains controversial, with over two-thirds of current evidence originating from US populations. This study evaluated AKI risk with VAN/PTZ vs VAN plus other beta-lactams (BLs) in Chinese patients, where VAN plus meropenem dominates empirical therapy. This study included patients receiving >48 h of VAN combined with a single BL. We compared AKI risk between VAN/PTZ and VAN/other-BLs. The primary outcome was incidence of stages 2–3 AKI, and the secondary outcome was major adverse kidney events at 60 days (MAKE_60_). Propensity score matching was used to match patients between comparison groups, and ORs were calculated. Among 8,460 screened patients, 5,632 were ultimately included: 279 received VAN/PTZ, 4,197 received VAN/carbapenems, and 1,156 received VAN/cephalosporins. Matched cohorts showed balanced demographics and clinical features. VAN/PTZ showed no elevated AKI risk when compared to VAN/other-BLs (OR = 1.20, 0.61–2.42), as well as compared to VAN/meropenem (OR = 1.13, 0.57–2.24) or VAN/cefepime (OR = 0.82, 0.34–1.96). Consistently, no elevated MAKE_60_ was observed when VAN/PTZ compared to VAN/other-BLs (OR = 1.20, 0.61–2.42), VAN/meropenem (OR = 1.24, 0.76–2.01), or VAN/cefepime (OR = 0.65, 0.28–1.47). This large multicenter cohort of Chinese patients demonstrated that VAN/PTZ was not associated with increased risk of AKI or MAKE_60_.

**IMPORTANCE:**

Acute kidney injury (AKI) associated with vancomycin (VAN) plus piperacillin-tazobactam (PTZ) remains controversial. Research evaluating this risk remains limited in Chinese cohorts. To the best of our knowledge, this multicenter retrospective cohort study is the largest study to date comparing the risk of AKI for VAN/PTZ against VAN plus other beta-lactams (BLs), VAN/meropenem (MEN), or VAN/cefepime (FEP) in the Chinese population. The study indicated that VAN/PTZ did not demonstrate an increased risk of stages 2–3 AKI and MAKE60 compared to VAN plus other BLs, as well as VAN/MEN, VAN/FEP. Our findings challenge common concerns about nephrotoxicity of VAN/PTZ mainly from Western populations and provide new safety evidence for clinical practice in China. While residual confounding inherent to observational designs remains a limitation, this study offers the highest-quality real-world evidence to date supporting non-inferior renal safety of VAN/PTZ relative to VAN plus other BLs in Asian populations.

## INTRODUCTION

Empiric antimicrobial therapy directed against both *Pseudomonas aeruginosa* and methicillin-resistant *Staphylococcus aureus* (MRSA) is recommended for the majority hospital-acquired infections in the United States (1). Vancomycin (VAN) combined with antipseudomonal beta-lactams (BLs) (e.g., piperacillin-tazobactam [PTZ], meropenem [MEN], cefepime [FEP], etc.) was considered standard antibiotics to cover MRSA and Gram-negative bacteria and infections, respectively (2). VAN use is often associated with nephrotoxicity (3–5). When considering the risk of acute kidney injury (AKI), VAN combined with PTZ (VAN/PTZ) has received the most attention, with studies reporting over a twofold increased AKI risk (6–10). However, this finding remains controversial with clinical evidence evolving. A key factor perpetuating this controversy is that ~90% of existing evidence originates from retrospective studies, which are inherently susceptible to confounding factors that may bias outcomes (7). Recent findings from the ACORN randomized controlled trial (RCT) revealed no increased AKI risk with PTZ vs FEP in patients predominantly receiving concomitant VAN (>75% of the cohort) (11, 12).

VAN and PTZ rank as the first and second most utilized antibiotics in US hospitals, followed by other top 10 BLs: ceftriaxone, cefazolin, FEP, and MEN (13). In contrast, VAN/PTZ combination usage was relatively uncommon in China, whereas VAN was frequently concomitant with carbapenems, comprising over 50% of the combination (predominantly MEN) (14–16). This clinical practice disparity stems from China’s universal penicillin skin testing requirement for all patients vs the US approach, where testing is reserved for higher-risk individuals with a history of recent or severe anaphylaxis (17, 18). Given the heavy workload of clinicians in China, physicians frequently favor prescribing BLs exempt from skin testing (cephalosporins and carbapenems) over agents requiring testing like PTZ when clinical indications are comparable (19, 20). Furthermore, in US healthcare settings, VAN and PTZ ranked among the most frequently implicated antibiotics in nephrotoxic exposures (21). Conversely, among hospitalized patients in China, furosemide was the predominant drug associated with AKI, followed by VAN, pantoprazole, mannitol, torsemide, spironolactone, and omeprazole (22).

Over two-thirds of current evidence on VAN/PTZ-associated AKI originates from the US populations, raising concerns about potential confounding from geographical selection bias (7). Research evaluating this risk remains limited in Chinese cohorts. To date, only two retrospective studies from China have examined VAN/PTZ-associated AKI, both reporting an increased risk compared with other beta-lactams (cefepime or carbapenems) (15, 16). However, the comparator beta-lactams examined in these studies were relatively narrow. Although Dong Wu et al. performed between-group balance comparisons and multivariable regression analyses, they did not employ propensity score matching (PSM); therefore, residual confounding could not be fully excluded. Notably, both VAN and PTZ bind to renal transporters that mediate creatinine secretion. A growing body of opinion and research suggests that mild AKI diagnosed based on SCr elevations could reflect a “pseudo-toxicity” phenomenon, mediated by effects on tubular secretion of creatinine without toxic effects on kidney parenchyma (6, 7, 10, 23, 24). Therefore, we conducted a large-scale multicenter retrospective cohort study in China. This study included all potential concomitant beta-lactam antibiotics and applied propensity score matching to minimize bias from retrospective confounding. We used a more stringent definition of AKI, defined as Kidney Disease: Improving Global Outcomes (KDIGO) stages 2 and 3 to more accurately capture true nephrotoxicity (10, 25). This study contributes new evidence to the growing body of literature. Additionally, these data from China, featuring prescribing patterns distinct from US hospitals, mitigate confounding from population selection bias due to geographical variations in clinical practice.

## MATERIALS AND METHODS

### Study design and settings

This was a multicenter retrospective cohort study conducted at three academic medical centers in Shanghai, China: Zhongshan Hospital, Fudan University; the Second Affiliated Hospital of Naval Medical University; and Tongji Hospital of Tongji University. The protocol has received approval from the Medical Ethics Committee of Zhongshan Hospital, Fudan University (B2024-451), with a waiver of informed consent. Ethics approvals were obtained from all participating sites. The study protocol was prospectively registered in the Chinese Clinical Trial Registry (ChiCTR2400094474). Data collection spanned January 2009 through December 2024, and the date were analyzed in January 2025.

### Patient selection

**Fig 1:**
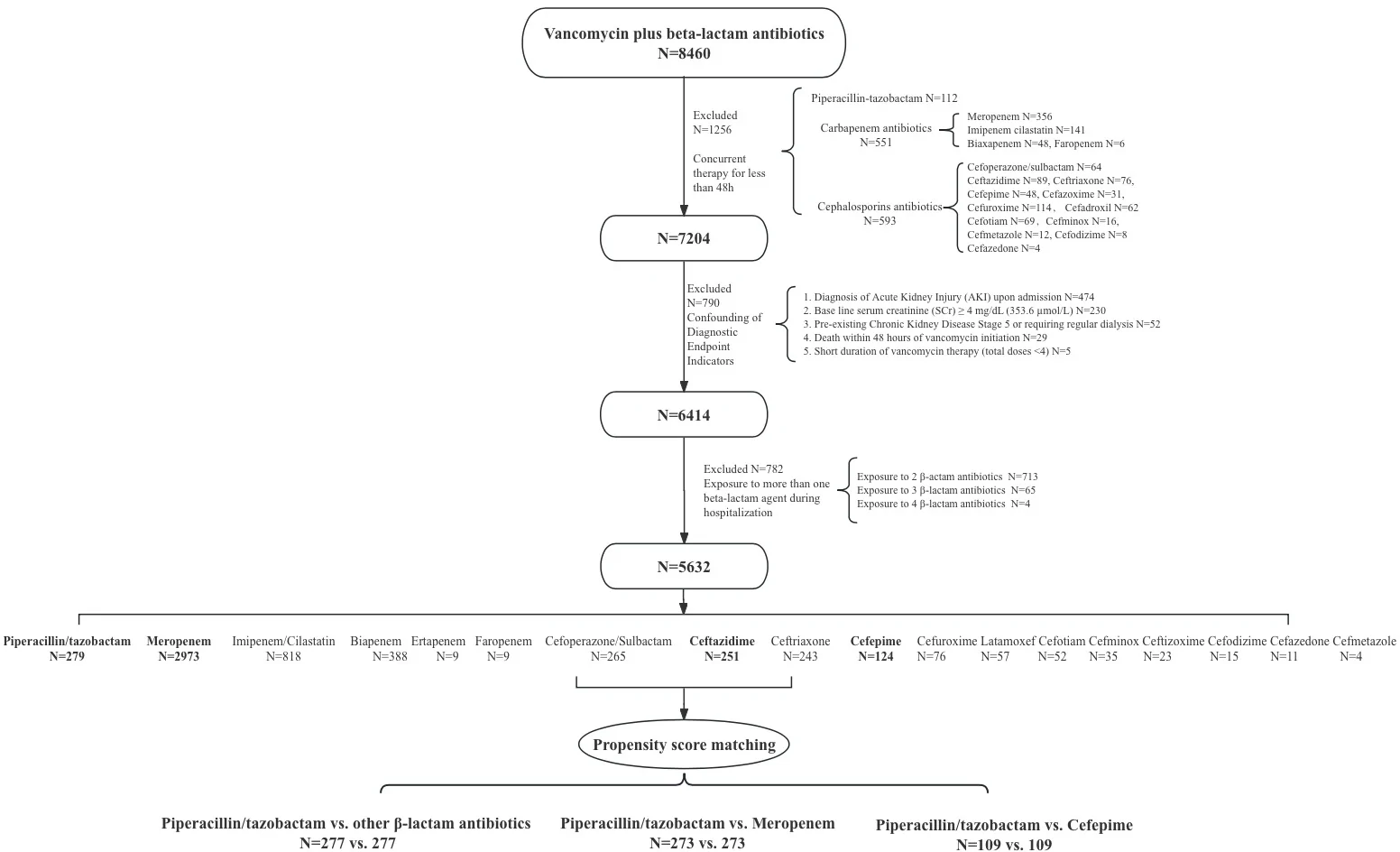
Study design and flowchart of patients.

Adult patients (≥18 years old) were included if they received continuous concurrent therapy with VAN and BLs for at least 48 h. The BLs comprised PTZ, carbapenems (MEN, imipenem/cilastatin, biapenem, ertapenem, faropenem), and cephalosporins (cefoperazone/sulbactam, ceftazidime, ceftriaxone, FEP, cefozopran, cefuroxime, latamoxef, cefotaxime, cefotiam, cefminox, cefdinir, and cefmetazole). We recorded the time interval between initiation of the two antibiotics. Concomitant therapy was defined as the two antibiotics having an overlapping administration period of at least 48 h, without requiring simultaneous initiation or termination. Their serum creatinine levels were measured at least once before and after medication administration, respectively. Patients were excluded if they were pregnant; if they received concurrent therapy for less than 48 h; if they received less than four doses of VAN, had stage 5 chronic kidney disease or were receiving regular dialysis, with baseline serum creatinine (SCr) was ≥4 mg/dL (353.6 μmolL), had AKI on admission, died within 48 h of VAN therapy initiation; if there was exposure to more than one beta-lactam agent during hospitalization (Fig. 1).

### Data collection and definitions

Data were extracted from electronic health records, including demographic information, comorbid conditions, pathophysiological parameters, laboratory values, concomitant medications, and other clinically relevant variables. Detailed information on the specific clinical variables collected can be seen in Table S1. Other BLs were defined as all BLs excluding PTZ. Baseline SCr was calculated as the median of all creatinine values in the 6 months preceding the index admission.

### End points

The primary outcome was the incidence of AKI, defined as Kidney Disease Improving Global Outcomes (KDIGO) 2012 guidelines stage 2 or 3 AKI (6, 7, 10, 23, 25). Specifically, stage 2 AKI was defined as an increase in SCr to 2.0–2.9 times baseline, and stage 3 AKI was defined as an increase in SCr to ≥3.0 times baseline, or an increase in SCr to ≥4.0 mg/dL (≥353.6 µmol/L), or initiation of renal replacement therapy (25). Therefore, this study only included patients whose serum creatinine levels were measured at least once both before and after medication administration, as this is an essential prerequisite for diagnosing AKI. Secondary outcomes included major acute kidney events at 60 days (MAKE_60_), dialysis rate, time to AKI onset, and 60-day mortality. MAKE_60_ was defined as a composite end point which included a persistent doubling of SCr level from baseline, a new requirement of renal replacement therapy, or death (9, 26).

### Statistical analysis

Continuous variables were summarized as means with standard deviation (SD) or medians with interquartile range (IQR) based on distribution normality. Categorical data were summarized as frequencies (percentages). Continuous data were compared across groups using analysis of variance or Kruskal-Wallis tests and categorical data using *χ*^2^ or Fisher exact tests. Logistic regression models were fit to compare the odds ratios of AKI and MAKE_60_ outcomes between VAN/PTZ and VAN plus other BLs.

PSM is a statistical method for causal inference amidst confounding factors (27). Propensity scores were derived through elastic net regression (*α* = 0.5, *λ* optimized via 5-fold cross-validation) incorporating 30 covariates covering demographics, comorbidities, nephrotoxic exposures, and illness severity, including age, gender, hypertension, diabetes, cerebral infarction, leukemia, lymphoma, metastatic cancer, renal insufficiency, hepatic insufficiency, cardiac insufficiency, hypoalbuminemia, multiple organ failure, sepsis, heart failure, shock, baseline SCr, VAN daily dose, combined with nephrotoxic drugs, number of combined nephrotoxic drugs, non-steroidal anti-inflammatory drugs (NSAIDs), vasopressors, loop diuretic, colistin, aminoglycoside, calcineurin inhibitors (CNIs), renin-angiotensin-aldosterone system inhibitors (RAASi), contrast agent, chemotherapeutic drugs, and proton pump inhibitor (PPI). Nephrotoxic agents include NSAIDs, vasopressors, loop diuretic, colistin, aminoglycoside, CNIs, RAASi, contrast agent, chemotherapeutic drugs, and PPI. Missing data (≤15% missingness) were addressed via multiple imputation using predictive mean matching (five imputations, 10 iterations). VAN plus other BLs and specific drugs of interest, including VAN/MEN and VAN/FEP, were compared in a 1:1 ratio with the group receiving VAN/PTZ. Separation issues were mitigated by iterative exclusion of perfectly predictive variables. Nearest-neighbor 1:1 matching without replacement used a caliper width of 0.2 standard deviations of the logit propensity score, with balance verified by standardized mean differences <0.1 and variance ratios between 0.8 and 1.25. Post-matching analyses applied generalized linear models (logit link) for binary outcomes (reported as adjusted odds ratios with 95% confidence intervals) and Wilcoxon rank-sum tests for continuous variables. Statistical significance was set at *P* < 0.05 (two-tailed).

For the primary outcome, we further conducted subgroup analyses as age (greater than or equal to 65 years old vs less than 65 years old), estimated glomerular filtration rate (eGFR) stratification (greater than or equal to 60 mL/min/1.73 m^2^ vs less than 60 mL/min/1.73 m^2^), illness severity (ICU vs non-ICU), vancomycin concentration (greater than or equal to 15 mg/L vs less than 15 mg/L), and vancomycin treatment duration stratification (greater than or equal to 14 days vs less than 14 days). Statistical analyses were conducted using RStudio software (version 4.2.1).

## RESULTS

### Baseline characteristics

A total of 8,460 patients who received VAN combined with BLs were screened (Fig. 1). The final cohort comprised 5,632 patients. The three participating centers enrolled 3,518, 1,510, and 604 patients, respectively. Of these, 279 (5.0%) received VAN/PTZ; 4,197 (74.5%) received VAN/carbapenems, including MEN (*N* = 2,973, 52.8%), imipenem/cilastatin (*N* = 818), etc., and 1,156 patients (20.5%) receiving VAN plus cephalosporins, including cefoperazone/sulbactam (*N* = 265), ceftazidime (*N* = 251), ceftriaxone (*N* = 243), etc. After PSM, among patients received VAN/PTZ, 64.6% (179/279) were male, with a median age of 63.0 years (IQR 51.0–73.0); in patients received VAN plus other BLs, 66.8% (185/277) were male, with a median age of 64.0 [54.0–72.0] (Table S1).

Table S1 presents the demographic and clinical characteristics of patients receiving VAN plus other BLs. Before PSM, the VAN/PTZ cohort demonstrated significantly older age, higher comorbidity burden (hypertension, diabetes, cerebral infarction, etc.), and greater illness severity (ICU admissions, septicemia, and heart failure prevalence) compared to the VAN plus other BLs group. Patients in the VAN/PTZ group had lower nephrotoxic agent exposure and reduced baseline SCr levels. After PSM, all characteristics achieved balance with standardized differences < 0.1 (Table S1). We further compared the highly concerned VAN-based antibiotic combinations (VAN/PTZ vs VAN/MEN or VAN/FEP). There were differences in demographic and clinical characteristics between the VAN/PTZ cohort and the patients receiving VAN/MEN (Table S2) or VAN/FEP (Table S3) before PSM, and these differences were no longer present after PSM.

### Risk of AKI between VAN-based antibiotic combination

**Fig 2:**
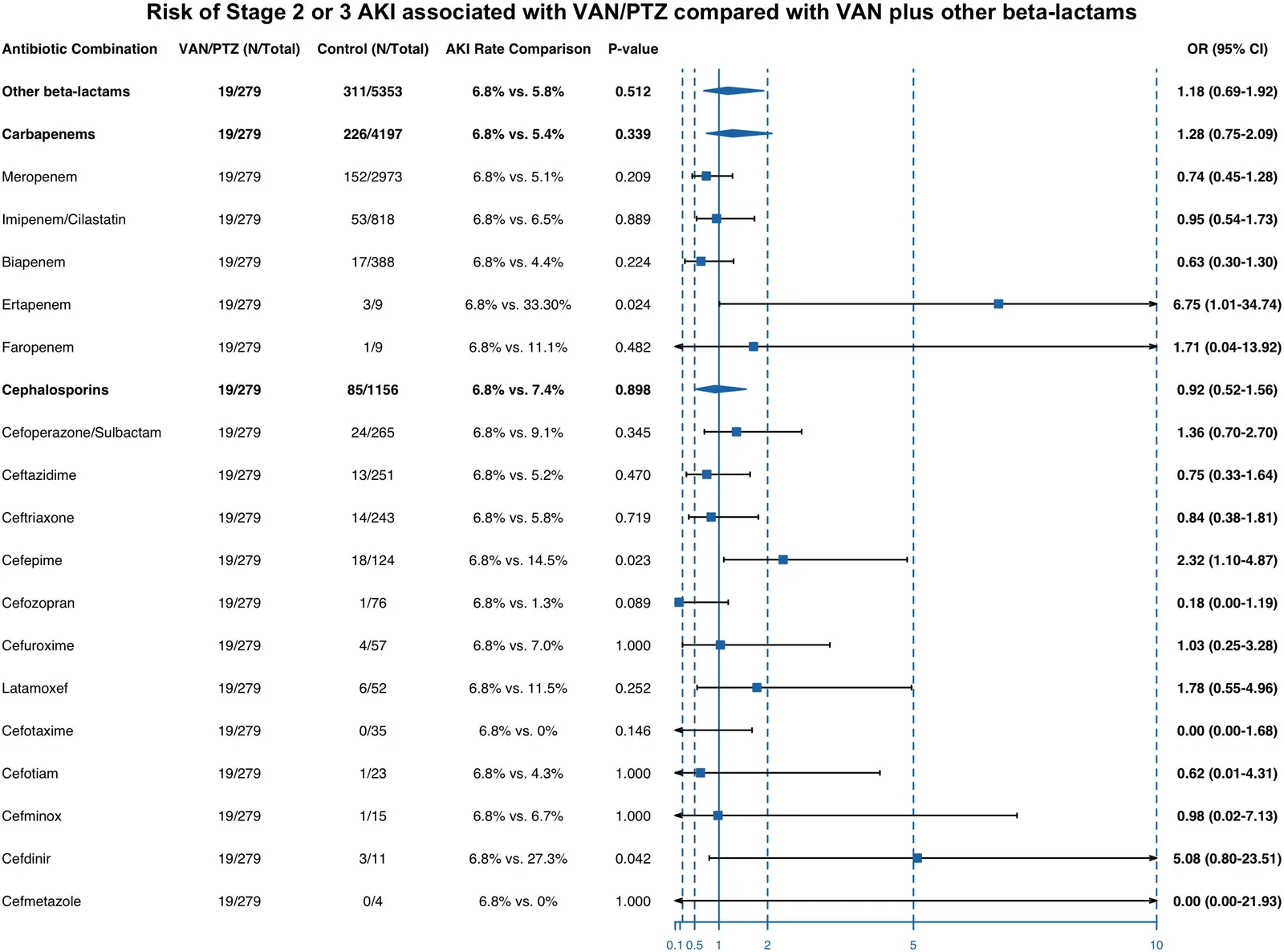
Risk of stage 2 or 3 AKI associated with VAN/PTZ compared with VAN plus other beta-lactams. Other beta-lactams were defined as all BLs excluding PTZ. AKI, acute kidney injury; BLs, beta-lactams; CI, confidence interval; OR, odds ratio; PTZ, piperacillin-tazobactam; VAN, vancomycin.

**Fig 3:**
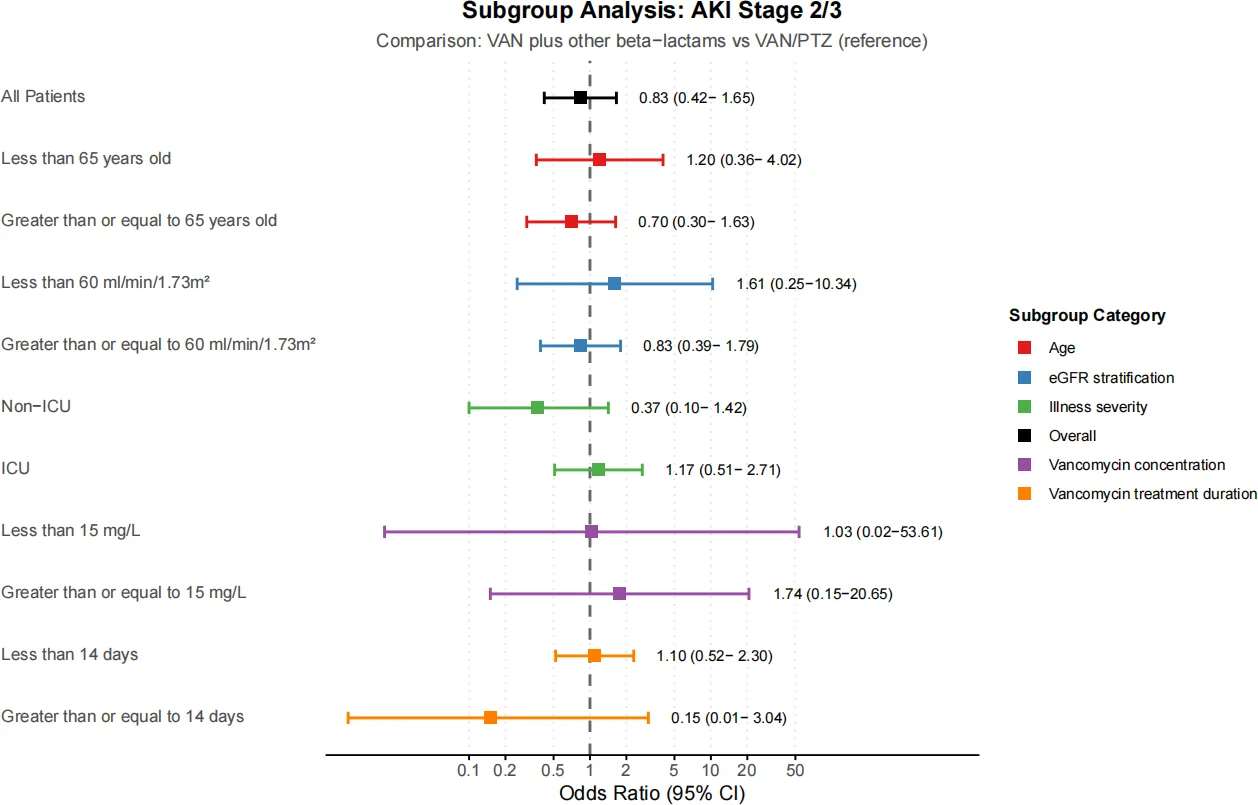
Subgroup analysis of risk of stage 2 or 3 AKI associated with VAN/PTZ compared with VAN plus other beta-lactams. Subgroups include age, eGFR, illness severity, vancomycin concentration, and treatment duration. Other beta-lactams were defined as all BLs excluding PTZ. AKI, acute kidney injury; BLs, beta-lactams; CI, confidence interval; eGFR, estimated glomerular filtration rate; OR, odds ratio; PTZ, piperacillin-tazobactam; VAN, vancomycin.

Figure 2 displays the risk of stage 2 or 3 AKI when comparing VAN/PTZ with VAN plus other BLs. Outcomes before and after PSM are detailed in Tables S1 to S3. The incidence of stage 2 or 3 AKI was 6.8% (19/279) among patients receiving VAN/PTZ. Unadjusted analyses demonstrated no increased risk of stage 2 or 3 AKI when VAN/PTZ was compared with VAN plus other BLs (OR = 1.18, 0.69–1.92), VAN plus carbapenems (OR = 1.28, 0.75–2.09), or VAN plus cephalosporins (OR = 0.92, 0.52–1.56) (Fig. 2). Post-PSM analyses similarly showed no elevated AKI risk for VAN/PTZ compared to VAN plus other BLs (OR = 1.20, 0.61–2.42) (Table 1). The results of subgroup analyses were consistent with this, and no significantly increased risk of AKI associated with VAN/PTZ was observed across all subgroups (age, eGFR, illness severity, vancomycin concentration, and treatment duration) (Fig. 3).

**TABLE 1:** Outcomes associated with VAN/PTZ compared to VAN plus other beta-lactams after propensity score matching^*b*^

Outcomes	VAN/PTZ *N* = 277	VAN/other BLs *N* = 277	OR (95% CI)	*P*
**AKI stage 2/3**	**19 (6.9)^*c*^**	**16 (5.8)**	**1.20 (0.61–2.42)**	**0.727**
**MAKE_60_ composite**	**42 (15.2)**	**29 (10.5)**	**1.53 (0.93–2.56)**	**0.127**
All-stage AKI	52 (18.8)	38 (13.7)	1.45 (0.92–2.31)	0.134
AKI severity				0.299
AKI stage 1	35 (12.6)	22 (7.9)	1.57 (0.89–2.80)	
AKI stage 2	10 (3.6)	8 (2.9)	1.30 (0.48–3.67)	
AKI stage 3	7 (2.5)	8 (2.9)	1.12 (0.44–2.85)	
Time to AKI (IQR)	4.00 (2.00, 7.25)	5.00 (3.00, 7.93)	/	0.549
AKI requiring dialysis	3 (1.1)	4 (1.4)	0.75 (0.15–3.42)	1.000
Death (within 60 days)	32 (11.6)	21 (7.6)	1.59 (0.90–2.87)	0.149
Hospital length of stay^*a*^	35.23 ± 28.66	43.38 ± 154.89	/	0.429
AKI recovery at discharge	16 (5.8)	11 (4)	0.68 (0.28–1.58)	0.431
AKI full-recovery	13 (4.7)	0 (4)	0.84 (0.33–2.07)	0.835
AKI part-recovery	3 (1.1)	0 (0)	0 (0–2.41)	0.249
Time to recovery	13.00 (21.91)	12.05 (9.39)	/	0.657

^*a*^ Data are presented as mean ± standard deviation.

^*b*^ AKI, acute kidney injury; 95% CI, 95% confidence interval; IQR, interquartile range; MAKE_60_, major acute kidney events at 60 days; OR, odds ratio; PTZ, piperacillin/tazobactam; VAN, vancomycin; /, not applicable.

^*c*^ Bold indicates the primary and key secondary composite outcomes.

For antibiotic combinations that are of much concern, the risk of stage 2 or 3 AK associated with VAN/PTZ was compared to VAN/MEN or VAN/FEP. In unadjusted analyses, VAN/PTZ showed no increased risk of AKI vs VAN/MEN (OR = 0.74, 0.45–1.28) but demonstrated 2.32-fold higher odds vs VAN/FEP (OR = 2.32, 1.10–4.87) (Fig. 2). After PSM, no increased AKI risk was observed when VAN/PTZ was compared with VAN/MEN (OR = 1.13, 0.57–2.24) (Table 2) or VAN/FEP (OR = 0.82, 0.34–1.96) (Table 3). Subgroup analyses remained consistent with the primary findings, demonstrating no significantly increased risk of AKI for VAN/PTZ relative to VAN/MEN (Fig. S1 or VAN/FEP (Fig. S2) across all subgroups.

**TABLE 2:** Outcomes associated with VAN/PTZ compared to VAN/MEN after propensity score matching^*b*^

Outcomes	VAN/PTZ *N* = 273	VAN/MEN *N* = 273	OR (95% CI)	*P*
**AKI stage 2/3**	**19 (7.0)^*c*^**	**17 (6.2)**	**1.13 (0.57–2.24)**	**0.863**
**MAKE_60_ composite**	**42 (15.4)**	**35 (12.8)**	**1.24 (0.76–2.01)**	**0.461**
All-stage AKI	52 (19.0)	42 (15.4)	1.29 (0.83–2.03)	0.308
AKI severity				0.570
AKI stage 1	35 (12.8)	25 (9.2)	1.36 (0.79–2.38)	
AKI stage 2	10 (3.7)	11 (4.0)	0.81 (0.32–1.99)	
AKI stage 3	7 (2.6)	6 (2.2)	1.69 (0.62–5.04)	
Time to AKI (IQR)	4.00 (2.0–8.03)	3.95 (2.53, 5.97)	/	0.630
AKI requiring dialysis	3 (1.1)	1 (0.4)	3.02 (0.38–61.31)	0.616
Death (within 60 days)	32 (11.7)	27 (9.9)	1.21 (0.70–2.09)	0.581
Hospital length of stay^*a*^	35.06 ± 28.80	32.58 ± 35.03	/	0.397
AKI recovery at discharge	17 (6.2)	12 (4.4)	0.69 (0.30–1.57)	0.446
AKI full-recovery	14 (5.1)	9 (3.3)	0.63 (0.24–1.60)	0.395
AKI part-recovery	3 (1.1)	3 (1.1)	1.00 (0.13–7.53)	1.000
Time to AKI recovery	11.01 (20.89)	8.47 (4.57)	/	0.079

^*a*^ Data are presented as mean ± standard deviation.

^*b*^ AKI, acute kidney injury; 95% CI, 95% confidence interval; IQR, interquartile range; MAKE_60_, major acute kidney events at 60 days; MEN, meropenem; OR, odds ratio; PTZ, piperacillin/tazobactam; VAN, vancomycin; /, not applicable.

^*c*^ Bold indicates the primary and key secondary composite outcomes.

**TABLE 3:** Outcomes associated with VAN/PTZ compared to VAN/FEP after propensity score matching^*a*^^,^^*b*^

Outcomes	VAN/PTZ *N* = 109	VAN/FEP *N* = 109	OR (95% CI)	*P*
**AKI stage 2/3**	**11 (10.1)^*c*^**	**16 (14.7)**	**0.65 (0.28–1.47)**	**0.411**
**MAKE_60_ composite**	22 (20.2)	22 (20.2)	1.00 (0.51–1.94)	1.000
All-stage AKI	24 (22.0)	24 (22.0)	1.00 (0.53–1.90)	1.000
AKI severity			/	0.549
AKI stage 1	14 (12.8)	9 (8.3)	1.71 (0.69–4.49)	
AKI stage 2	5 (4.6)	8 (7.3)	0.48 (0.13–1.58)	
AKI stage 3	5 (4.6)	7 (6.4)	0.87 (0.29–2.50)	
Time to AKI (IQR)	3.50 (2.00–7.00)	6.91 (2.98–12.88)		0.105
AKI requiring dialysis	2 (1.8)	2 (1.8)	1.00 (0.12–8.46)	1.000
Death (within 60 days)	17 (15.6)	11 (10.1)	1.65 (0.74–3.80)	0.311
Hospital length of stay	38.57 ± 33.85	37.40 ± 29.91	/	0.803
AKI recovery at discharge	6 (5.5)	5 (6.4)	1.18 (0.33–4.40)	1.000
AKI full-recovery	4 (3.7)	5 (4.6)	1.26 (0.26–6.54)	1.000
AKI part-recovery	2 (1.8)	2 (1.8)	1.00 (0.07–14.02)	1.000
Time to AKI recovery	13.42 (17.87)	11.01 (12.38)	/	0.695

^*a*^ Data are presented as mean ± standard deviation.

^*b*^ AKI, acute kidney injury; 95% CI, 95% confidence interval; FEP, cefepime; IQR, interquartile range; MAKE_60_, major acute kidney events at 60 days; OR, odds ratio; PTZ, piperacillin/tazobactam; VAN, vancomycin; /, not applicable.

^*c*^ Bold indicates the primary and key secondary composite outcomes.

The stratified Kaplan-Meier survival analysis of time-to-AKI indicated no difference when VAN/PTZ was compared with VAN/other BLs (*P* = 0.72) (Fig. S3), VAN/MEN (*P* = 0.84) (Fig. S4), or VAN/FEP (*P* = 0.30) (Fig. S5) (Fig. 3).

### Secondary end points

**Fig 4:**
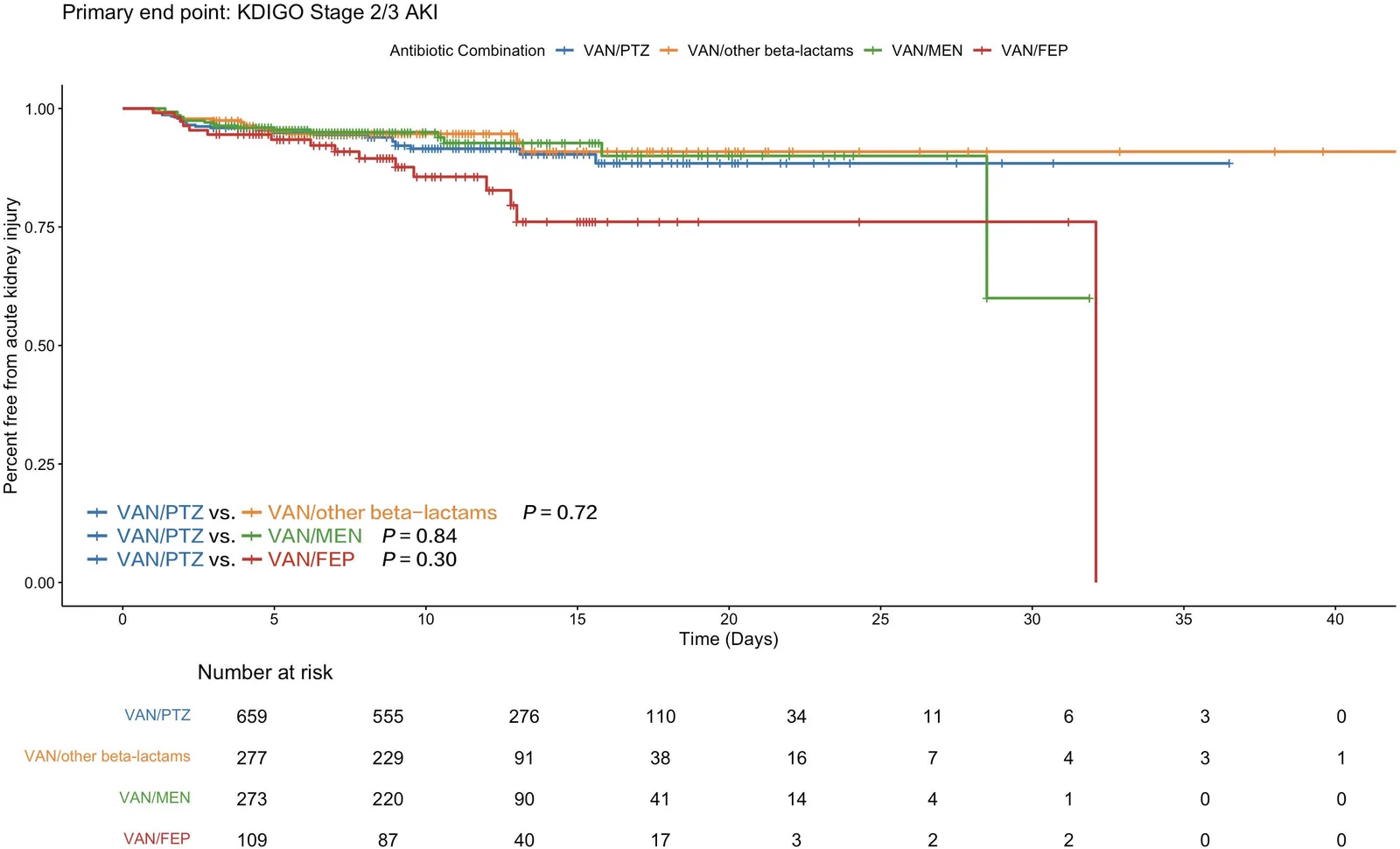
Kaplan-Meier analysis of time to AKI for VAN/PTZ and VAN/other-BLs, VAN/MEN, or VAN/FEP. VAN plus other BLs and specific drugs of interest, including VAN/MEN and VAN/FEP were compared in a 1:1 ratio with the group receiving VAN/PTZ. The number of VAN/PTZ is shown as the sum of the number of multiple comparisons. Time to AKI was measured from the start of combination therapy. Survival estimate was illustrated until the outcome (AKI) occurred or until they were censored. The table below the curves provides the estimated survival probabilities (i.e., AKI-free rates) at selected time points (days 5, 10, 15, 20, 25, 30, 35, and 40) for each comparison. The log-rank test *P* values are displayed on each curve (*P* = 0.72, 0.84, and 0.30, respectively), indicating no significant difference in time to AKI between VAN/PTZ and VAN/MEN or VAN/FEP. The steep decline in the VAN/FEP curve at day 32 occurs because only 2 patients remained at risk at that time and one of them developed AKI, causing a 50% drop in the estimated AKI-free survival. This is a statistical artifact due to the very small sample size in the tail of the curve and does not represent a true increase in AKI risk; the log-rank test (*P* = 0.30) confirms no overall difference between VAN/PTZ and VAN/FEP. Other BLs were defined as all BLs excluding PTZ. Time to AKI was measured from the combination start time until 48 h after combination discontinuation. AKI, acute kidney injury; BLs, beta-lactams; FEP, cefepime; KDIGO, kidney disease improving global outcomes; MEN, meropenem; PTZ, piperacillin/tazobactam; VAN, vancomycin.

**Fig 5:**
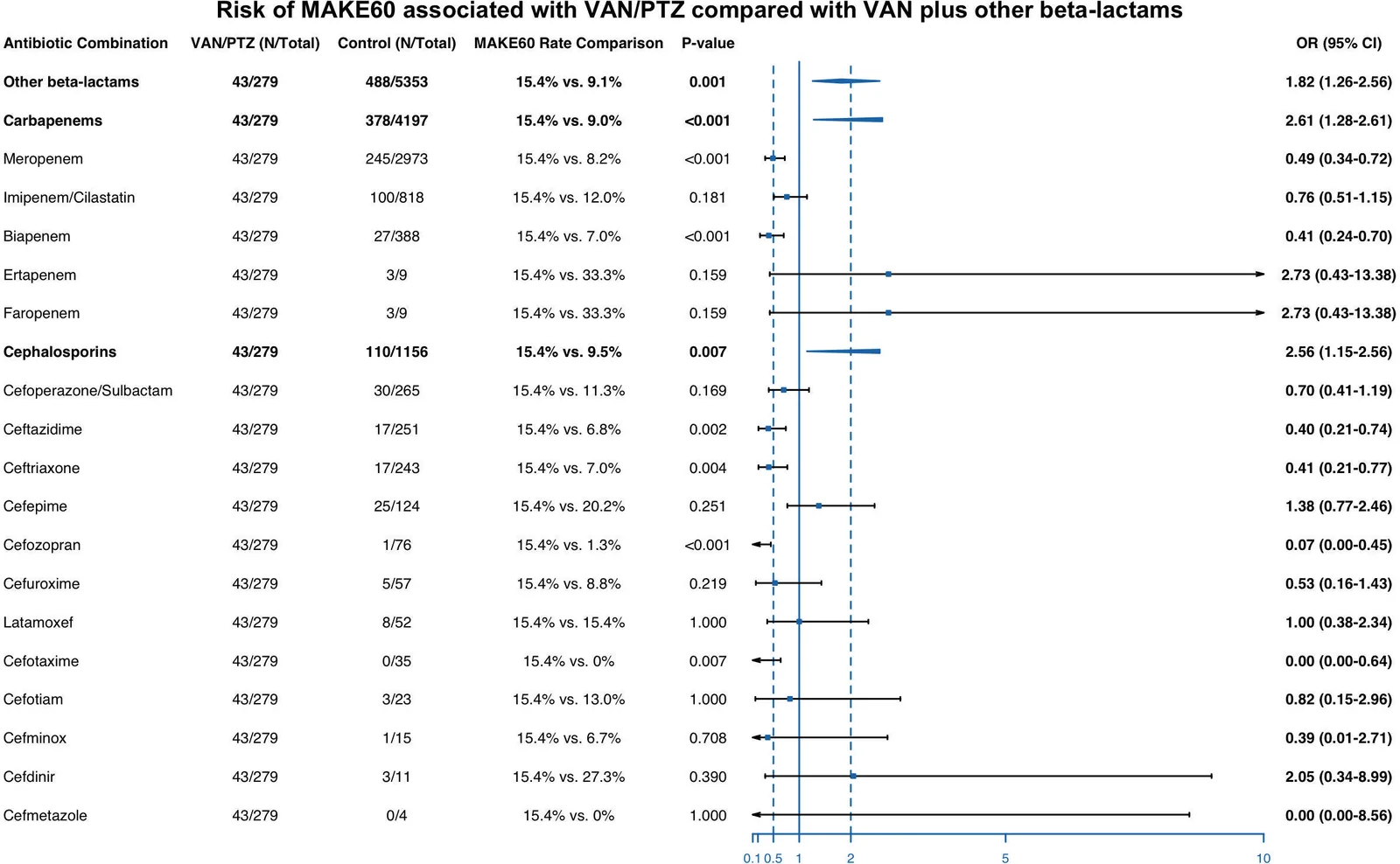
Risk of MAKE60 associated with VAN/PTZ compared with VAN plus other beta-lactams. MAKE60 included a persistent doubling of serum creatinine level from baseline, a new requirement of renal replacement therapy, or death at 60 days. Other beta-lactams were defined as all BLs excluding PTZ. AKI, acute kidney injury; BLs, beta-lactams; CI, confidence interval; MAKE60, major acute kidney events at 60 days; OR, odds ratio; PTZ, piperacillin-tazobactam; VAN, vancomycin.

Figure 4 displays the risk of MAKE_60_ when comparing VAN/PTZ with VAN plus other BLs. In unadjusted analyses, the rate of MAKE_60_ was higher among patients receiving VAN/PTZ compared with those receiving VAN plus other BLs (OR = 1.82, 1.26–2.56), VAN plus carbapenems (OR = 2.61, 1.28–2.61), or VAN plus cephalosporins (OR = 2.56, 1.15–2.56) (Fig. 5). After PSM, no significant risk of MAKE_60_ was observed when comparing VAN/PTZ with VAN plus other beta-lactams (OR = 1.53, 0.93–2.56). Similarly, no significant differences were observed for dialysis requirement or 60-day mortality when comparing VAN/PTZ with VAN plus other BLs. (Table 1).

For antibiotic combinations that are of much concern, in unadjusted analyses, VAN/PTZ was associated with a lower risk of MAKE_60_ when compared to VAN/MEN (OR = 0.49, 0.34–0.72) and no increased risk when compared to VAN/FEP (OR = 1.38, 0.77–2.46) (Fig. 5). After PSM, no significant difference in the risk of MAKE_60_ was observed when VAN/PTZ was compared with VAN/MEN (OR = 1.24, 0.76–2.01) (Table 2) or VAN/FEP (OR = 0.65, 0.28–1.47) (Table 3). Similarly, no significant differences were observed for dialysis requirement or 60-day mortality when comparing VAN/PTZ with VAN/MEN (Table 2) or VAN/FEP (Table 3).

## DISCUSSION

To the best of our knowledge, this multicenter retrospective cohort study is the largest study to date comparing the risk of AKI for VAN/PTZ against VAN plus other BLs, VAN/MEN, or VAN/MEP in the Chinese population. The study indicated that VAN/PTZ did not demonstrate an increased risk of stages 2–3 AKI and MAKE_60_ compared to VAN plus other BLs, as well as VAN/MEN, VAN/FEP.

One characteristic of VAN-based antibiotic regimens in Chinese populations is that the most common BLs concomitant with VAN are MEN, rather than PTZ, which reflects differences in prescribing patterns compared to US hospitals (13–16). The persistent controversy regarding whether VAN/PTZ confers increased nephrotoxicity stems largely from retrospective evidence vulnerable to confounding (7). As noted earlier, existing publications in this domain are largely derived from US populations; our study addresses this bias by providing high-quality data from China (13). Two previous small-scale retrospective observational studies have investigated the risk of AKI associated with VAN/PTZ vs VAN plus other BLs in Chinese cohorts (15, 16). Kang Liu et al. reported increased AKI risk with VAN/PTZ but no elevated requirement for renal replacement therapy (15). Similarly, Dong Wu et al. reported increased AKI incidence but no increased in in-hospital mortality with this combination (16). Dong Wu et al. performed between-group balance analysis (showing no statistically significant baseline differences) and multivariable regression, which adjusted for several confounders; nonetheless, residual confounding may still have affected the estimates. Moreover, neither study assessed severe AKI (stages 2–3) using the KDIGO criteria. Mild AKI diagnosed based on SCr elevations could reflect a “pseudo-toxicity” phenomenon arising from piperacillin’s inhibition of tubular creatinine secretion (6, 23). In this study, the primary outcome was defined as stage 2 or 3 AKI, which is a higher degree of creatinine elevation than that expected from secretion inhibition alone and, therefore, suggests true kidney injury (10). Our findings support that VAN/PTZ was not associated with increased risk of AKI, aligning with recent results from the ACORN RCT conducted by Qian et al. (11). Acute interstitial nephritis (AIN) is an important type of drug-induced AKI, and its pathogenesis involves a T cell-mediated immune response (28). BLs may cause AIN via delayed-type hypersensitivity (type IV reaction) (29). In addition, although the risk of cross reactivity between penicillins and cephalosporins remains debated, some patients with penicillin allergy may have an increased risk of reacting to cephalosporins (30). The clinical practice regarding penicillin skin testing policy in China, compared with that in the United States, likely results in a lower proportion of patients receiving vancomycin who have true penicillin allergy (17, 18, 20). A positive penicillin skin test may not be directly causative of VAN/PTZ-associated AKI; however, this selection bias could potentially lower the observed AKI risk associated with VAN/PTZ in Chinese cohorts.

Death was the primary driver of the secondary composite outcome MAKE₆₀. It is improbable that all deaths were attributable to AKI, as evidenced by comparable incidences of stages 2–3 AKI and new renal replacement therapy requirements across treatment groups. Before PSM, patients receiving VAN/PTZ had worse comorbidities (higher prevalence of hypertension, diabetes, and hematologic malignancies) and greater illness severity (higher ICU admission rates and hypoalbuminemia), with significantly higher risk of MAKE₆₀ compared to VAN plus other BLs, VAN/MEN, and VAN plus biapenem. After PSM, no statistically significant differences existed in comorbidities or illness severity between VAN/PTZ and comparator groups, and results demonstrated no elevated MAKE₆₀ risk. We contend that the observed elevation in MAKE₆₀ risk likely reflects greater underlying disease severity among decedents.

This study conducted a comprehensive comparative analysis of AKI risk between VAN/PTZ and VAN concomitant with each of 16 other BLs. To our knowledge, this appears to be the most extensive analysis of BLs diversity to date in such comparative nephrotoxicity research (2). Based on a comprehensive literature review, Alosaimy et al. proposed that hydrophobic BLs with high OAT-3 affinity potentiate VAN nephrotoxicity, as measured by SCr elevations (31). This study contributes clinical data to this conjecture. Clinical realities are inherently multifaceted; although clinical outcomes diverge from theoretical conjectures, such discrepancies nevertheless progress us toward refined mechanistic insights. To reduce confounding bias, stringent inclusion/exclusion criteria were applied. Particularly, we excluded patients exposed to ≥2 BLs during their entire VAN treatment course. Current research typically groups patients by initial antibiotic regimens, without tracking subsequent BLs exposures during VAN therapy (8, 10, 11). By analyzing exposures to all classes of BLs and implementing rigorous exclusion criteria, this study guaranteed statistical independence across comparator groups.

PSM can minimize the discrepancies between the different groups of patients and enable balanced and unbiased comparison (7, 27). Through systematic matching of potential confounders impacting endpoint assessments, we minimized bias in outcome evaluation. We observed significant differences in clinical characteristics between VAN/PTZ and other VAN-based antibiotic regimens prior to PSM. Before PSM, discordant outcomes existed between comparator groups: AKI risk (primary outcome) and MAKE₆₀ risk (secondary outcome) demonstrated inconsistent directional trends. Contrary to prevailing evidence, VAN/PTZ even demonstrated significantly lower AKI risk compared to VAN/FEP, contradicting most published studies (7, 11, 31). After PSM, which achieved balanced baseline characteristics between comparator groups, both AKI and MAKE₆₀ outcomes consistently demonstrated comparable risk profiles for VAN/PTZ vs alternative regimens, thus reducing the concern of VAN/PTZ-associated AKI. When RCT evidence remains inadequate, PSM, which emulates randomization principles, establishes that VPT imposes no excess AKI risk relative to VAN plus other BLs.

VAN/FEP and VAN/MEN are the scrutinized antibiotic combinations beyond VAN/PTZ (7, 8, 32–34). In our analysis, prior to matching, VAN/PTZ showed no increased risk of AKI compared to VAN/MEN and was associated with an even lower AKI risk compared to VAN/FEP. After PSM, VAN/PTZ was not associated with an increased risk of AKI compared to either VAN/FEP or VAN/MEN, nor with an increased risk of MAKE_60_. After minimizing confounding bias through PSM, the findings of this study are consistent with that of recent RCT (11, 23). Our study adds to this growing body of literature by comparing VAN/PTZ with VAN plus other BL in a multicenter Chinese population.

This study has several strengths. First, this represents the largest multicenter cohort study to date investigating the risk of AKI associated with VAN/PTZ in Chinese patients. Second, our analysis encompasses the broadest range of BLs in published literature. The exclusion criterion prohibiting multiple BLs exposures during VAN treatment ensures independence between comparator groups. Third, after adjusting for confounding factors, the concordant pre-/post-PSM results suggest VAN/PTZ confers no higher AKI risk than VAN plus other BLs. This study also has several limitations. First, with the observational and retrospective nature of this study, some residual confounding is possible. We collected covariates as comprehensive as possible and used PSM to control for confounding factors. Second, our definition of AKI did not include the urine output of the KDIGO criteria. This limitation stems from incomplete data documentation in retrospective records, and some patients such as non-critically ill patients do not record urine volume. Third, as in the literature, the factor of duration of combination therapy between VAN and different BLs was not analyzed in this study. Fourth, we did not exclude patients who had received any of the study antibiotics within 7 days prior to the combination therapy, which may introduce some residual confounding. However, based on previous evidence, adjusting for prior antibiotic exposure as a covariate is unlikely to materially change the results, as the increased risk is mainly attributed to the concurrent use of the two antibiotics. Also, the exclusion criteria already included: if there was exposure to more than one beta-lactam agent during hospitalization, which helped to minimize the confounding.

### Conclusion

In conclusion, this large multicenter cohort study of Chinese patients demonstrated that VAN/PTZ was not associated with increased risk of AKI or MAKE_60_ compared to VAN plus other BLs or specific regimens including VAN/MEN and VAN/FEP. Our findings challenge common concerns about nephrotoxicity of VAN/PTZ mainly from Western populations and provide new safety evidence for clinical practice in China. While residual confounding inherent to observational designs remains a limitation, this study offers the highest quality real-world evidence to date supporting non-inferior renal safety of VAN/PTZ relative to VAN plus other BLs in Asian populations. Future high-quality evidence from RCTs remains essential to provide definitive confirmation.

## Data Availability

The data that support the findings of this study are available from the corresponding author upon reasonable request.

## AFTER EPUB

*[This article was published on 6 July 2026 with an error in Figure 1. The Figure 1 was corrected in the current version, posted on 17 September 2026.]*

## SUPPLEMENTAL MATERIAL

Tables S1 to S3; Figures S1 to S5 spectrum.00821-26-s0001.docx Table S1. The demographic, clinical characteristics and outcomes of patients receiving vancomycin plus beta-lactam antibiotics. Table S2. The demographic, clinical characteristics and outcomes of patients receiving vancomycin plus piperacillin/tazobactam vs vancomycin plus meropenem. Table S3. The demographic, clinical characteristics and outcomes of patients receiving vancomycin plus piperacillin/tazobactam vs vancomycin plus cefepime Figure S1. Subgroup analysis of Risk of stage 2 or 3 AKI associated with VAN/PTZ compared with VAN plus MEN. Subgroups include age, eGFR, illness severity, vancomycin concentration, and treatment duration. Abbreviations: AKI, acute kidney injury; CI, confidence interval; eGFR, estimated glomerular filtration rate; MEN, meropenem; OR, odds ratio; PTZ, piperacillin-tazobactam; VAN, Vancomycin. Figure S2. Subgroup analysis of Risk of stage 2 or 3 AKI associated with VAN/PTZ compared with VAN plus FEP. Subgroups include age, eGFR, illness severity, vancomycin concentration, and treatment duration. Abbreviations: AKI, acute kidney injury; CI, confidence interval; eGFR, estimated glomerular filtration rate; FEP, cefepime; OR, odds ratio; PTZ, piperacillin-tazobactam; VAN, Vancomycin. Figure S3. Kaplan-Meier analysis of time to AKI for VAN/TZP and VAN/other-BLs. Other BLs were defined as all BLs excluding PTZ. Time to AKI was measured from the combination start time until 48 hours after combination discontinuation. Abbreviations: AKI, acute kidney injury; BLs, beta-lactams; KDIGO, Kidney Disease Improving Global Outcomes; PTZ, piperacillin/tazobactam; VAN, Vancomycin. Figure S4. Kaplan-Meier analysis of time to AKI for VAN/TZP and VAN/MEN. Time to AKI was measured from the combination start time until 48 hours after combination discontinuation. Abbreviations: AKI, acute kidney injury; KDIGO, Kidney Disease Improving Global Outcomes; MEN, meropenem; PTZ, piperacillin/tazobactam; VAN, Vancomycin. Figure S5. Kaplan-Meier analysis of time to AKI for VAN/TZP and VAN/FEP. Time to AKI was measured from the combination start time until 48 hours after combination discontinuation. The sharp decline in the VAN/FEP curve around day 32 occurs because only 2 patients remained at risk at that time and one of them developed AKI, causing a 50% drop in the estimated AKI‑free survival. This is a statistical artifact due to the very small sample size in the tail of the curve and does not represent a true increase in AKI risk. Abbreviations: AKI, acute kidney injury; FEP, cefepime; KDIGO, Kidney Disease Improving Global Outcomes; PTZ, piperacillin/tazobactam; VAN, Vancomycin.

Graphical Abstract spectrum.00821-26-s0002.tif Graphical Abstract

ASM does not own the copyrights to Supplemental Material that may be linked to, or accessed through, an article. The authors have granted ASM a non-exclusive, world-wide license to publish the Supplemental Material files. Please contact the corresponding author directly for reuse.
