# Supplementary material for: Risk of acute kidney injury in patients on concomitant vancomycin and piperacillin-tazobactam compared to vancomycin and other beta-lactam agents: a large multicenter retrospective cohort study from China
Source: Microbiol Spectr. 2026 Jul 6;14(10):e00821-26. doi: 10.1128/spectrum.00821-26 (PMC13689445; doi:10.1128/spectrum.00821-26)
Supplement: Supplementary file 1 [file spectrum.00821-26-s0001.docx]

Supplementary Material

Supplementary Table 1. The demographic, clinical characteristics and outcomes of patients receiving vancomycin plus beta-lactam antibiotics.

|  | Before PSM | | | After PSM | | |
| --- | --- | --- | --- | --- | --- | --- |
|  | BETA N=5353 | PTZ N =279 | P | BETA N=277 | PTZ N=277 | P |
| Demographic information | | | | | | |
| Gender (Male) | 3449 (64.4) | 181 (64.9) | 0.931 | 185 (66.8) | 179 (64.6) | 0.655 |
| Age, years (IQR) | 60.0 [48.0,69.0] | 63.0 [51.0,73.0] | <0.001 | 64.0 [54.0,72.0] | 63.0 [51.0,73.0] | 0.739 |
| BMI, kg/m² (IQR)^a^ | 23.7 [21.3,26.1] | 23.2 [20.9,26.5] | 0.995 | 24.3 [22.0,27.0] | 23.2 [20.9,26.5] | 0.421 |
| Concomitant underlying diseases | | | | | | |
| Hypertension | 1361 (25.4) | 96 (34.4) | 0.001 | 96 (34.7) | 94 (33.9) | 0.929 |
| Diabetes | 626 (11.7) | 64 (22.9) | <0.001 | 65 (23.5) | 63 (22.7) | 0.920 |
| Coronary artery disease | 563 (10.5) | 41 (14.7) | 0.036 | 46 (16.6) | 40 (14.4) | 0.557 |
| Atrial fibrillation | 380 (7.1) | 22 (7.9) | 0.705 | 21 (7.6) | 22 (7.9) | 1.000 |
| Cerebral infarction | 364 (6.8) | 31 (11.1) | 0.009 | 32 (11.6) | 31 (11.2) | 1.000 |
| Hyperlipidaemia | 55 (1.0) | 8 (2.9) | 0.011 | 6 (2.2) | 8 (2.9) | 0.787 |
| Metastatic cancer | 784 (14.6) | 38 (13.6) | 0.728^e^ | 34 (12.3) | 38 (13.7) | 0.705^e^ |
| Leukemia | 266 (5.0) | 4 (1.4) | 0.004^e^ | 7 (2.5) | 4 (1.4) | 0.545^e^ |
| Lymphoma | 403 (7.5) | 2 (0.7) | <0.001^e^ | 1 (0.4) | 2 (0.7) | 1.000^e^ |
| Renal insufficiency | 164 (3.1) | 12 (4.3) | 0.326 | 13 (4.7) | 12 (4.3) | 1.000 |
| Chronic Kidney Disease |  |  | 0.903 |  |  | 0.432 |
| Stage 1 | 3030 (59.4) | 165 (62.3) |  | 156 (58.0) | 164 (62.4) |  |
| Stage 2 | 1394 (27.3) | 67 (25.3) |  | 82 (30.5) | 67 (25.5) |  |
| Stage 3 | 551 (10.8) | 26 (9.8) |  | 28 (10.4) | 25 (9.5) |  |
| Stage 4 | 123 (2.3) | 7 (2.5) |  | 3 (1.1) | 7 (2.5) |  |
| Hepatic insufficiency | 320 (6.0) | 22 (7.9) | 0.241 | 26 (9.4) | 22 (7.9) | 0.650 |
| Cardiac insufficiency |  |  | <0.001 |  |  | 0.063 |
| Grade 0 | 4703 (87.9) | 237 (84.9) |  | 234 (84.5) | 236 (85.2) |  |
| Grade 1 | 312 (5.8) | 11 (3.9) |  | 22 (7.9) | 11 (4.0) |  |
| Grade 2 | 51 (1.0) | 8 (2.9) |  | 2 (0.7) | 8 (2.9) |  |
| Grade 3 | 224 (4.2) | 11 (3.9) |  | 13 (4.7) | 11 (4.0) |  |
| Grade 4 | 63 (1.2) | 12 (4.3) |  | 6 (2.2) | 11 (4.0) |  |
| Severity of illness | | | | | | |
| Admission to ICU | 2046 (38.2) | 156 (55.9) | <0.001 | 157 (56.7) | 154 (55.6) | 0.864 |
| Septicopyemia | 967 (18.1) | 28 (10.0) | 0.001 | 28 (10.1) | 28 (10.1) | 1.000 |
| Heart failure | 71 (1.3) | 9 (3.2) | 0.019 | 7 (2.5) | 8 (2.9) | 1.000 |
| Shock | 182 (3.4) | 10 (3.6) | 1.000 | 13 (4.7) | 10 (3.6) | 0.670 |
| Hypoalbuminemia | 2258 (42.2) | 167 (59.9) | <0.001 | 158 (57.0) | 165 (59.6) | 0.605 |
| Concomitant nephrotoxic drugs | | | | | | |
| Concomitant nephrotoxic drugs | 2150 (40.2) | 88 (31.5) | 0.005 | 73 (26.4) | 86 (31.0) | 0.260 |
| Numbers of nephrotoxic drugs |  |  | 0.005 |  |  | 0.054 |
| 0 | 3203 (59.8) | 191 (68.5) |  | 204 (73.6) | 191 (69.0) |  |
| 1 | 1566 (29.3) | 76 (27.2) |  | 51 (18.4) | 74 (26.7) |  |
| 2 | 501 (9.4) | 10 (3.6) |  | 19 (6.9) | 10 (3.6) |  |
| 3 | 78 (1.5) | 2 (0.7) |  | 3 (1.1) | 2 (0.7) |  |
| 4 | 5 (0.1) | 0 (0.0) |  | 0 | 0 |  |
| Aminoglycoside | 60 (1.1) | 11 (3.9) | 0.001^e^ | 8 (2.9) | 10 (3.6) | 0.811^e^ |
| Contrast agent | 97 (1.8) | 2 (0.7) | 0.261 | 1 (0.4) | 2 (0.7) | 1.000 |
| Vasopressors | 335 (6.3) | 4 (1.4) | 0.002 | 4 (1.4) | 4 (1.4) | 1.000 |
| loop diuretic | 1674 (31.3) | 55 (19.7) | <0.001 | 42 (15.2) | 55 (19.9) | 0.180 |
| CNIs | 90 (1.7) | 1 (0.4) | 0.178^e^ | 2 (0.7) | 1 (0.4) | 1.000^e^ |
| RAASi | 385 (7.2) | 23 (8.2) | 0.672 | 30 (10.8) | 22 (7.9) | 0.302 |
| NSAIDs | 129 (2.4) | 6 (2.2) | 0.940 | 6 (2.2) | 6 (2.2) | 1.000 |
| PPI | 2953 (55.2) | 131 (47.0) | 0.009 | 128 (46.2) | 131 (47.3) | 0.865 |
| Vancomycin daily dose |  |  | 0.013 |  |  | 0.778 |
| ≤ 1g | 1285 (24.0) | 88 (31.5) |  | 79 (28.5) | 86 (31.0) |  |
| > 1g, ≤ 2g | 3564 (66.6) | 164 (58.8) |  | 168 (60.6) | 164 (59.2) |  |
| > 2g | 504 (9.4) | 27 (9.7) |  | 30 (10.8) | 27 (9.7) |  |
| Laboratory tests | | | | | | |
| Baseline SCr, μmol/L (IQR) | 68.0 [54.0,87.0] | 65.0 [52.0,81.5] | 0.037 | 65.0 [53.0,86.0] | 65.0 [52.0,81.0] | 0.732 |
| Baseline eGFR, ml/min/1.73m² (IQR) | 95.4 [76.1,109.2] | 97.6 [73.3,108.5] | 0.791 | 94.4 [77.4,107.3] | 97.6 [73.4,108.0] | 0.485 |
| Serum albumin, g/L (IQR) | 35.0 [30.0,39.0] | 32.0 [27.9,36.2] | <0.001 | 33.0 [28.2,37.0] | 32.0 [27.9,36.2] | 0.644 |
| Outcomes | | | | | | |
| Maximum-stage AKI | 695 (13.0) | 53 (19.0) | 0.005 | 38 (13.7) | 52 (18.8) | 0.134 |
| Time to AKI (IQR) | 4.62 [2.14,7.92] | 4.95 [2.41,8.10] | 0.949 | 4.20 [2.00,7.22] | 4.90 [2.35,8.10] | 0.781 |
| AKI any stage |  |  | 0.006 |  |  | 0.299 |
| 1 | 390 (7.3) | 36 (12.9) |  | 22 (7.9) | 35 (12.6) |  |
| 2 | 165 (3.1) | 10 (3.6) |  | 8 (2.9) | 10 (3.6) |  |
| 3 | 140 (2.6) | 7 (2.5) |  | 8 (2.9) | 7 (2.5) |  |
| AKI stage 2/3 | 311 (5.8) | 19 (6.8) | 0.574 | 16 (5.8) | 19 (6.9) | 0.727 |
| AKI requiring dialysis | 26 (0.5) | 3 (1.1) | 0.362 | 4 (1.4) | 3 (1.1) | 1 |
| Death (within 60 d) | 303 (5.7) | 33 (11.8) | <0.001 | 21 (7.6) | 32 (11.6) | 0.149 |
| MAKE60 composite | 488 (9.1) | 43 (15.4) | 0.001 | 29 (10.5) | 42 (15.2) | 0.127 |
| Hospital length of staya | 31.84 ± 84.39 | 35.58 ± 28.81 | 0.497 | 43.38 ± 154.89 | 35.23 ± 28.66 | 0.429 |
| AKI recovery at discharge | 290 (5.4) | 28 (10.0) | 0.002 | 17 (6.1) | 27 (9.7) | 0.157 |
| AKI Full-recovery | 200 (3.7) | 14 (5.0) | 0.352 | 11 (4.0) | 13 (4.7) | 0.835 |
| AKI Part-recovery | 235 (4.4) | 17 (6.1) | 0.233 | 10 (3.6) | 16 (5.8) | 0.315 |

Abbreviations: BMI, body mass index; BETA, beta-lactam antibiotics; CNIs, calcineurin inhibitors, including cyclosporine, tacrolimus; eGFR, estimated glomerular filtration rate; ICU, intensive care unit; IQR, interquartile range; NSAIDs, non-steroidal anti-inflammatory drugs; PTZ, piperacillin/tazobactam; PPI, proton pump inhibitor; PSM, propensity score matching; RAASi, renin-angiotensin-aldosterone system inhibitors; SCr, serum creatinine.

Data are presented as No. (%) or median (IQR); Renal insufficiency defined as eGFR<60 ml/min/1.73m².

^a^ More than 20% of the values were missing.

^e^ Refers to the calibration of the Fisher's Exact Test.

Supplementary Table 2. The demographic, clinical characteristics and outcomes of patients receiving vancomycin plus piperacillin/tazobactam vs vancomycin plus meropenem

|  | Before PSM | | | After PSM | | |
| --- | --- | --- | --- | --- | --- | --- |
|  | MEN N=2973 | PTZ N =279 | P | MEN N=273 | PTZ N=273 | P |
| Demographic information | | | | | | |
| Gender (Male) | 1968 (66.2) | 181 (64.9) | 0.704 | 173 (63.4) | 177 (64.8) | 0.789 |
| Age, years (IQR) | 60.00 [48.00,68.00] | 63.00 [51.00,73.00] | 0.001 | 62.00 [52.00,71.00] | 63.00 [51.00,73.00] | 0.964 |
| BMI, kg/m² (IQR)^a^ | 23.88 [21.55,26.35] | 23.15 [20.91,26.50] | 0.642 | 24.16 [21.40,26.69] | 23.15 [20.91,26.50] | 0.672 |
| Concomitant underlying diseases | | | | | | |
| Hypertension | 764 (25.7) | 96 (34.4) | 0.002 | 88 (32.2) | 92 (33.7) | 0.785 |
| Diabetes | 340 (11.4) | 64 (22.9) | <0.001 | 64 (23.4) | 62 (22.7) | 0.919 |
| Cerebral infarction | 196 (6.6) | 31 (11.1) | 0.007 | 34 (12.5) | 30 (11.0) | 0.690 |
| Coronary artery disease | 373 (12.5) | 41 (14.7) | 0.349 | 43 (15.8) | 40 (14.7) | 0.812 |
| Hyperlipidaemia | 40 (1.3) | 8 (2.9) | 0.079 | 5 (1.8) | 8 (2.9) | 0.575 |
| Atrial fibrillation | 266 (8.9) | 22 (7.9) | 0.626 | 23 (8.4) | 21 (7.7) | 0.875 |
| Leukemia | 158 (5.3) | 4 (1.4) | 0.002 | 5 (1.8) | 4 (1.5) | 1.000^e^ |
| Lymphoma | 301 (10.1) | 2 (0.7) | <0.001 | 4 (1.5) | 2 (0.7) | 0.686^e^ |
| Metastatic cancer | 423 (14.2) | 38 (13.6) | 0.858 | 40 (14.7) | 38 (13.9) | 0.903^e^ |
| Renal insufficiency | 92 (3.1) | 12 (4.3) | 0.359 | 12 (4.4) | 12 (4.4) | 1.000 |
| Chronic Kidney Disease |  |  | 0.261 |  |  | 0.298 |
| Stage 1 | 1600 (55.6) | 165 (62.3) |  | 164 (62.4) | 162 (62.5) |  |
| Stage 2 | 823 (28.6) | 67 (25.3) |  | 71 (27.0) | 65 (25.1) |  |
| Stage 3 | 384 (13.3) | 26 (9.8) |  | 27 (10.3) | 25 (9.7) |  |
| Stage 4 | 72 (2.5) | 7 (2.5) |  | 1 (0.4) | 7 (2.5) |  |
| Hepatic insufficiency | 199 (6.7) | 22 (7.9) | 0.527 | 24 (8.8) | 22 (8.1) | 0.878 |
| Cardiac insufficiency |  |  | <0.001 |  |  | 0.021 |
| Grade 0 | 2519 (84.7) | 237 (84.9) |  | 235 (86.1) | 232 (85.0) |  |
| Grade 1 | 231 (7.8) | 11 (3.9) |  | 21 (7.7) | 11 (4.0) |  |
| Grade 2 | 38 (1.3) | 8 (2.9) |  | 1 (0.4) | 8 (2.9) |  |
| Grade 3 | 149 (5.0) | 11 (3.9) |  | 11 (4.0) | 10 (3.7) |  |
| Grade 4 | 36 (1.2) | 12 (4.3) |  | 5 (1.8) | 12 (4.4) |  |
| Severity of illness | | | | | | |
| Admission to ICU | 1121 (37.7) | 156 (55.9) | <0.001 | 152 (55.7) | 151 (55.3) | 1.000 |
| Septicopyemia | 651 (21.9) | 28 (10.0) | <0.001 | 28 (10.3) | 28 (10.3) | 1.000 |
| Heart failure | 48 (1.6) | 9 (3.2) | 0.085 | 8 (2.9) | 9 (3.3) | 1.000 |
| Shock | 102 (3.4) | 10 (3.6) | 1.000 | 21 (7.7) | 10 (3.7) | 0.064 |
| Hypoalbuminemia | 1127 (37.9) | 167 (59.9) | <0.001 | 167 (61.2) | 161 (59.0) | 0.662 |
| Concomitant nephrotoxic drugs | | | | | | |
| Concomitant nephrotoxic drugs | 1393 (46.9) | 88 (31.5) | <0.001 | 74 (27.1) | 85 (31.1) | 0.346 |
| Numbers of nephrotoxic drugs |  |  | <0.001 |  |  |  |
| 0 | 1580 (53.1) | 191 (68.5) |  | 199 (72.9) | 188 (68.9) | 0.369 |
| 1 | 976 (32.8) | 76 (27.2) |  | 57 (20.9) | 73 (26.7) |  |
| 2 | 347 (11.7) | 10 (3.6) |  | 14 (5.1) | 10 (3.7) |  |
| 3 | 65 (2.2) | 2 (0.7) |  | 3 (1.1) | 2 (0.7) |  |
| 4 | 5 (0.2) | 0 (0.0) |  | 0 | 0 |  |
| Aminoglycoside | 26 (0.9) | 11 (3.9) | <0.001 | 6 (2.2) | 8 (2.9) | 0.788^e^ |
| Contrast agent | 52 (1.7) | 2 (0.7) | 0.296 | 2 (0.7) | 2 (0.7) | 1.000 |
| Vasopressors | 245 (8.2) | 4 (1.4) | <0.001 | 7 (2.6) | 4 (1.5) | 0.542 |
| loop diuretic | 1116 (37.5) | 55 (19.7) | <0.001 | 53 (19.4) | 55 (20.1) | 0.914 |
| CNIs | 56 (1.8) | 1 (0.4) | 0.165^e^ | 1 (0.4) | 1 (0.4) | 1.000^e^ |
| RAASi | 256 (8.6) | 23 (8.2) | 0.772 | 19 (7.0) | 23 (8.4) | 0.63 |
| NSAIDs | 102 (3.4) | 6 (2.2) | 0.334 | 5 (1.8) | 6 (2.2) | 1.000 |
| PPI | 1778 (59.8) | 131 (47.0) | <0.001 | 133 (48.7) | 131 (48.0) | 0.932 |
| Vancomycin daily dose |  |  | <0.001 |  |  | 0.360 |
| ≤ 1g | 492 (16.5) | 88 (31.5) |  | 78 (28.6) | 83 (30.4) |  |
| > 1g, ≤ 2g | 2188 (73.6) | 164 (58.8) |  | 176 (64.5) | 163 (59.7) |  |
| > 2g | 293 (9.9) | 27 (9.7) |  | 19 (7.0) | 27 (9.9) |  |
| Laboratory tests | | | | | | |
| Baseline SCr, μmol/L (IQR) | 70.00 [55.00,91.00] | 65.00 [50.50,81.50] | <0.001 | 63.00 [51.00,77.00] | 65.00 [51.00,82.00] | 0.568 |
| Baseline eGFR, ml/min/1.73m² (IQR) | 93.56 [72.30,107.22] | 97.57 [73.32,108.48] | 0.114 | 96.95 [82.01,108.92] | 97.57 [73.37,108.03] | 0.554 |
| Serum albumin, g/L (IQR) | 35.50 [31.30,39.00] | 32.00 [27.85,36.15] | <0.001 | 32.00 [28.00,36.00] | 32.00 [28.10,36.30] | 0.613 |
| Outcomes | | | | | | |
| Maximum-stage AKI | 383 (12.9) | 53 (19.0) | 0.006 | 42 (15.4) | 52 (19.0) | 0.308 |
| Time to AKI (IQR) | 4.50 [2.48,7.97] | 4.00 [2.00,8.00] | 0.858 | 3.95 [2.53,5.97] | 4.00 [2.00,8.03] | 0.63 |
| AKI any stage |  |  | 0.024 |  |  | 0.57 |
| 1 | 235 (7.9) | 36 (12.9) |  | 25 (9.2) | 35 (12.8) |  |
| 2 | 83 (2.8) | 10 (3.6) |  | 11 (4.0) | 10 (3.7) |  |
| 3 | 65 (2.2) | 7 (2.5) |  | 6 (2.2) | 7 (2.6) |  |
| AKI stage 2/3 | 152 (5.1) | 19 (6.8) | 0.283 | 17 (6.2) | 19 (7.0) | 0.863 |
| AKI requiring dialysis | 10 (0.3) | 3 (1.1) | 0.169 | 1 (0.4) | 3 (1.1) | 0.616 |
| Death (within 60 d) | 150 (5.0) | 33 (11.8) | <0.001 | 27 (9.9) | 32 (11.7) | 0.581 |
| MAKE60 composite | 245 (8.2) | 43 (15.4) | <0.001 | 35 (12.8) | 42 (15.4) | 0.461 |
| Hospital length of stay | 30.27 ± 87.71 | 35.58 ± 28.81 | 0.354 | 32.58 ± 35.03 | 35.06 ± 28.80 | 0.397 |
| AKI recovery at discharge | 166 (5.6) | 28 (10.0) | 0.004 | 16 (5.9) | 27 (9.9) | 0.112 |
| AKI Full-recovery | 118 (4.0) | 14 (5.0) | 0.49 | 9 (3.3) | 14 (5.1) | 0.394 |
| AKI Part-recovery | 136 (4.6) | 17 (6.1) | 0.318 | 12 (4.4) | 17 (6.2) | 0.445 |

Abbreviations: BMI, body mass index; CNIs, calcineurin inhibitors, including cyclosporine, tacrolimus; eGFR, estimated glomerular filtration rate; ICU, intensive care unit; IQR, interquartile range; MEN, meropenem; NSAIDs, non-steroidal anti-inflammatory drugs; PTZ, piperacillin/tazobactam; PPI, proton pump inhibitor; PSM, propensity score matching; RAASi, renin-angiotensin-aldosterone system inhibitors; SCr, serum creatinine.

Data are presented as No. (%) or median (IQR); Renal insufficiency defined as eGFR<60 ml/min/1.73m².

^a^ More than 20% of the values were missing.

^e^ Refers to the calibration of the Fisher's Exact Test.

Supplementary Table 3. The demographic, clinical characteristics and outcomes of patients receiving vancomycin plus piperacillin/tazobactam vs vancomycin plus cefepime

|  | Before PSM | | | After PSM | | |
| --- | --- | --- | --- | --- | --- | --- |
|  | FEP N=124 | PTZ N =279 | P | FEP N=109 | PTZ N=109 | P |
| Demographic information | | | | | | |
| Gender (Male) | 84 (67.7) | 181 (64.9) | 0.655 | 71 (65.1) | 60 (55.0) | 0.167 |
| Age, years (IQR) | 63.00 [53.75,70.00] | 63.00 [51.00,73.00] | 0.788 | 64.00 [53.00,72.00] | 62.00 [51.00,73.00] | 0.935 |
| BMI, kg/m² (IQR)^a^ | 23.04 [20.26,25.54] | 23.15 [20.91,26.50] | 0.495 | 23.05 [20.32,25.49] | 24.03 [22.24,24.45] | 0.983 |
| Concomitant underlying diseases | | | | | | |
| Hypertension | 44 (35.5) | 96 (34.4) | 0.924 | 42 (38.5) | 31 (28.4) | 0.151 |
| Diabetes | 23 (18.5) | 64 (22.9) | 0.391 | 20 (18.3) | 23 (21.1) | 0.734 |
| Cerebral infarction | 11 (8.9) | 31 (11.1) | 0.615 | 10 (9.2) | 14 (12.8) | 0.516 |
| Coronary artery disease | 12 (9.7) | 41 (14.7) | 0.224 | 12 (11.0) | 16 (14.7) | 0.544 |
| Hyperlipidaemia | 0 (0.0) | 8 (2.9) | 0.129 | 0 (0.0) | 8 (7.3) | 0.012 |
| Atrial fibrillation | 6 (4.8) | 22 (7.9) | 0.369 | 5 (4.6) | 10 (9.2) | 0.284 |
| Leukemia | 2 (1.6) | 4 (1.4) | 1.000^e^ | 2 (1.8) | 2 (1.8) | 1.000^e^ |
| Lymphoma | 11 (8.9) | 2 (0.7) | <0.001^e^ | 2 (1.8) | 2 (1.8) | 1.000^e^ |
| Metastatic cancer | 32 (25.8) | 38 (13.6) | 0.004^e^ | 24 (22.0) | 21 (19.3) | 0.738^e^ |
| Renal insufficiency | 7 (5.6) | 12 (4.3) | 0.739 | 7 (6.4) | 8 (7.3) | 1.000 |
| Chronic Kidney Disease |  |  | 0.174 |  |  | 0.069 |
| Stage 1 | 70 (58.3) | 165 (62.3) |  | 60 (57.1) | 68 (66.7) |  |
| Stage 2 | 40 (33.3) | 67 (25.3) |  | 35 (33.3) | 21 (20.6) |  |
| Stage 3 | 7 (5.8) | 26 (9.8) |  | 7 (6.7) | 8 (7.8) |  |
| Stage 4 | 3 (2.5) | 7 (2.5) |  | 3 (2.9) | 5 (4.9) |  |
| Hepatic insufficiency | 14 (11.3) | 22 (7.9) | 0.359 | 13 (11.9) | 12 (11.0) | 1.000 |
| Cardiac insufficiency |  |  | 0.037 |  |  | 0.175 |
| Grade 0 | 115 (92.7) | 237 (84.9) |  | 100 (91.7) | 93 (85.3) |  |
| Grade 1 | 6 (4.8) | 11 (3.9) |  | 6 (5.5) | 5 (4.6) |  |
| Grade 2 | 0 (0.0) | 8 (2.9) |  | 0 (0.0) | 4 (3.7) |  |
| Grade 3 | 3 (2.4) | 11 (3.9) |  | 3 (2.8) | 6 (5.5) |  |
| Grade 4 | 0 (0.0) | 12 (4.3) |  | 0 (0.0) | 1 (0.9) |  |
| Severity of illness | | | | | | |
| Admission to ICU | 70 (56.5) | 156 (55.9) | 1.000 | 60 (55.0) | 68 (62.4) | 0.336 |
| Septicopyemia | 22 (17.7) | 28 (10.0) | 0.045 | 19 (17.4) | 18 (16.5) | 1.000 |
| Shock | 3 (2.4) | 10 (3.6) | 0.760 | 3 (2.8) | 5 (4.6) | 0.719 |
| Hypoalbuminemia | 70 (56.5) | 167 (59.9) | 0.595 | 62 (56.9) | 66 (60.6) | 0.680 |
| Concomitant nephrotoxic drugs | | | | | | |
| Concomitant nephrotoxic drugs | 56 (45.2) | 88 (31.5) | 0.012 | 47 (43.1) | 47 (43.1) | 1.000 |
| Numbers of nephrotoxic drugs |  |  | 0.004 |  |  | 0.239 |
| 0 | 68 (54.8) | 191 (68.5) |  | 62 (56.9) | 62 (56.9) |  |
| 1 | 40 (32.3) | 76 (27.2) |  | 35 (32.1) | 39 (35.8) |  |
| 2 | 15 (12.1) | 10 (3.6) |  | 12 (11.0) | 6 (5.5) |  |
| 3 | 1 (0.8) | 2 (0.7) |  | 0 (0.0) | 2 (1.8) |  |
| Aminoglycoside | 4 (3.2) | 11 (3.9) | 1.000^e^ | 4 (3.7) | 5 (4.6) | 1.000^e^ |
| Contrast agent | 3 (2.4) | 2 (0.7) | 0.348 | 3 (2.8) | 0 (0.0) | 0.245 |
| Vasopressors | 6 (4.8) | 4 (1.4) | 0.093 | 3 (2.8) | 3 (2.8) | 1.000 |
| loop diuretic | 41 (33.1) | 55 (19.7) | 0.005 | 34 (31.2) | 34 (31.2) | 1.000 |
| CNIs | 5 (4.0) | 1 (0.4) | 0.012^e^ | 2 (1.8) | 1 (0.9) | 1.000^e^ |
| RAASi | 10 (8.1) | 23 (8.2) | 1.000 | 9 (8.3) | 10 (9.2) | 1.000 |
| NSAIDs | 3 (2.4) | 6 (2.2) | 1.000 | 3 (2.8) | 4 (3.7) | 1.000 |
| PPI | 71 (57.3) | 131 (47.0) | 0.072 | 61 (56.0) | 48 (44.0) | 0.104 |
| Vancomycin daily dose |  |  | 0.861 |  |  | 0.533 |
| ≤ 1g | 37 (29.8) | 88 (31.5) |  | 33 (30.3) | 39 (35.8) |  |
| > 1g, ≤ 2g | 73 (58.9) | 164 (58.8) |  | 63 (57.8) | 61 (56.0) |  |
| > 2g | 14 (11.3) | 27 (9.7) |  | 13 (11.9) | 9 (8.3) |  |
| Laboratory tests | | | | | | |
| Baseline SCr, μmol/L (IQR) | 65.50 [53.08,85.00] | 64.00 [50.00,80.50] | 0.374 | 66.00 [53.00,85.00] | 54.00 [42.00,76.00] | 0.017 |
| Baseline eGFR, ml/min/1.73m² (IQR) | 93.54 [79.88,104.88] | 97.57 [73.32,108.48] | 0.797 | 93.18 [79.02,104.07] | 99.89 [74.55,111.82] | 0.179 |
| Serum albumin, g/L (IQR) | 32.05 [29.00,37.00] | 32.00 [27.85,36.15] | 0.606 | 32.00 [29.10,37.00] | 31.95 [27.78,36.00] | 0.393 |
| Outcomes | | | | | | |
| Maximum-stage AKI | 27 (21.8) | 53 (19.0) | 0.61 | 27 (21.8) | 53 (19.0) | 0.61 |
| Time to AKI (IQR) | 5.94 [2.47,11.27] | 4.00 [2.00,8.00] | 0.387 | 5.94 [2.47,11.27] | 4.00 [2.00,8.00] | 0.387 |
| AKI any stage |  |  | 0.048 |  |  | 0.048 |
| 1 | 10 (8.1) | 36 (12.9) |  | 10 (8.1) | 36 (12.9) |  |
| 2 | 9 (7.3) | 10 (3.6) |  | 9 (7.3) | 10 (3.6) |  |
| 3 | 8 (6.5) | 7 (2.5) |  | 8 (6.5) | 7 (2.5) |  |
| AKI stage 2/3 | 18 (14.5) | 19 (6.8) | 0.022 | 18 (14.5) | 19 (6.8) | 0.022 |
| AKI requiring dialysis | 2 (1.6) | 3 (1.1) | 1 | 2 (1.6) | 3 (1.1) | 1 |
| Death (within 60 d) | 13 (10.5) | 33 (11.8) | 0.824 | 13 (10.5) | 33 (11.8) | 0.824 |
| MAKE60 composite | 25 (20.2) | 43 (15.4) | 0.303 | 25 (20.2) | 43 (15.4) | 0.303 |
| Hospital length of staya | 35.90 ± 29.06 | 35.58 ± 28.81 | 0.925 | 35.90 ± 29.06 | 35.58 ± 28.81 | 0.925 |
| AKI recovery at discharge | 13 (10.5) | 28 (10.0) | 1 | 13 (10.5) | 28 (10.0) | 1 |
| AKI Full-recovery | 7 (5.6) | 14 (5.0) | 0.985 | 7 (5.6) | 14 (5.0) | 0.985 |
| AKI Part-recovery | 9 (7.3) | 17 (6.1) | 0.826 | 9 (7.3) | 17 (6.1) | 0.826 |

Abbreviations: BMI, body mass index; CNIs, calcineurin inhibitors, including cyclosporine, tacrolimus; eGFR, estimated glomerular filtration rate; FEP, cefepime; ICU, intensive care unit; IQR, interquartile range; NSAIDs, non-steroidal anti-inflammatory drugs; PPI, proton pump inhibitor; PTZ, piperacillin/tazobactam; PSM, propensity score matching; RAASi, renin-angiotensin-aldosterone system inhibitors; SCr, serum creatinine.

Data are presented as No. (%) or median (IQR); Renal insufficiency defined as eGFR<60 ml/min/1.73m².

^a^ More than 20% of the values were missing.

^e^ Refers to the calibration of the Fisher's Exact Test.

Supplementary Figure 1. Subgroup analysis of Risk of Stage 2 or 3 AKI associated with VAN/PTZ compared with VAN plus MEN. Subgroups include age, eGFR, illness severity, vancomycin concentration, and treatment duration. Abbreviations: AKI, acute kidney injury; CI, confidence interval; eGFR, estimated glomerular filtration rate; MEN, meropenem; OR, odds ratio; PTZ, piperacillin-tazobactam; VAN, Vancomycin.

Supplementary Figure 2. Subgroup analysis of Risk of Stage 2 or 3 AKI associated with VAN/PTZ compared with VAN plus FEP. Subgroups include age, eGFR, illness severity, vancomycin concentration, and treatment duration. Abbreviations: AKI, acute kidney injury; CI, confidence interval; eGFR, estimated glomerular filtration rate; FEP, cefepime; OR, odds ratio; PTZ, piperacillin-tazobactam; VAN, Vancomycin.

Supplementary Figure 3. Kaplan-Meier analysis of time to AKI for VAN/TZP and VAN/other-BLs. Other BLs were defined as all BLs excluding PTZ. Time to AKI was measured from the combination start time until 48 hours after combination discontinuation. Abbreviations: AKI, acute kidney injury; BLs, beta-lactams; KDIGO, Kidney Disease Improving Global Outcomes; PTZ, piperacillin/tazobactam; VAN, Vancomycin.

Supplementary Figure 4. Kaplan-Meier analysis of time to AKI for VAN/TZP and VAN/MEN. Time to AKI was measured from the combination start time until 48 hours after combination discontinuation. Abbreviations: AKI, acute kidney injury; KDIGO, Kidney Disease Improving Global Outcomes; MEN, meropenem; PTZ, piperacillin/tazobactam; VAN, Vancomycin.

Supplementary Figure 5. Kaplan-Meier analysis of time to AKI for VAN/TZP and VAN/FEP. Time to AKI was measured from the combination start time until 48 hours after combination discontinuation. The sharp decline in the VAN/FEP curve around day 32 occurs because only 2 patients remained at risk at that time and one of them developed AKI, causing a 50% drop in the estimated AKI‑free survival. This is a statistical artifact due to the very small sample size in the tail of the curve and does not represent a true increase in AKI risk. Abbreviations: AKI, acute kidney injury; FEP, cefepime; KDIGO, Kidney Disease Improving Global Outcomes; PTZ, piperacillin/tazobactam; VAN, Vancomycin.
